# Physical activity and mental health in school-aged children: a prospective two-wave study during the easing of the COVID-19 restrictions

**DOI:** 10.1186/s13034-023-00695-8

**Published:** 2024-01-03

**Authors:** Philipp M. Kopp, Eva Möhler, Peter Gröpel

**Affiliations:** 1 Saarland University Hospital (UKS), Homburg, Germany; 2 University of Vienna, Vienna, Austria; 3 Clinic for Child and Adolescent Psychiatry, Psychosomatics and Psychotherapy, Saarland University Hospital (UKS), Saarland University, Saarbrücken, Germany; 4 Division of Sport Psychology, Centre for Sport Science and University Sports, University of Vienna, Vienna, Austria

**Keywords:** COVID-19, Mental health, Sport, Exercise, School

## Abstract

**Background:**

Because physical activity (PA) has many benefits for children’s and adolescents’ mental health, it has been suggested that PA may be an effective strategy to physically and mentally recover from the COVID-19 pandemic. This study tested the reciprocal relationship between PA and mental health during the easing of COVID-19 restrictions. It was hypothesized that mental health during the pandemic would determine how much children and adolescents re-engage in PA after easing the restrictions. Furthermore, it was hypothesized that PA engagement would predict mental health improvement after the pandemic.

**Methods:**

This was a prospective study with two measurement occasions. Pretest data collection was undertaken in February 2022, shortly before German authorities eased and lifted the COVID-19 restrictions. The follow-up (posttest) occurred six weeks later (April 2022). Both times, a sample of elementary and grammar school students aged 6 to 18 years (*N* = 170) reported their mental health problems and health-related quality of life. Mental health problems were assessed with the Strengths and Difficulties Questionnaire and health-related quality of life was assessed with the KIDSCREEN-52 questionnaire. PA was measured continuously during the study period using smart electronic devices with a built-in pedometer.

**Results:**

PA gradually increased after the easing of COVID-19 restrictions (*p* < .001). This increase was unrelated to pretest mental health problems and health-related quality of life except for emotional symptoms (*p* = .041). Participants with higher emotional symptoms showed a sharper increase in PA towards the end of the study period. Furthermore, hyperactivity decreased (*p* = .004) and physical well-being (*p* = .004), perceived autonomy (*p* < .001), and perceived quality of school environment (*p* = .008) improved from before to after the easing of restrictions, yet participants’ PA predicted none of these changes.

**Conclusions:**

The adverse effects of COVID-19 containment on PA seem to alleviate after children and adolescents are allowed to return to schools. This is likely to be due to the school setting, which provides many different opportunities for formal and informal PA rather than students’ mental health. School-related PA programs should be part of children’s and adolescents’ recovery from the pandemic .

**Supplementary Information:**

The online version contains supplementary material available at 10.1186/s13034-023-00695-8.

## Background

Regular physical activity (PA) has many benefits for children’s and adolescents’ mental health [1, 2]. Physically active individuals typically report higher well-being [3], self-esteem [4], and health-related quality of life [5], whereas low levels of PA have been associated with increased risks of depression [6], anxiety [7], and psychological distress [8]. In clinical research, PA interventions in children and adolescent samples resulted in a significant reduction of depressive and posttraumatic stress symptoms in the target groups [1, 9], with effects comparable to psychotherapy and antidepressants [10–12]. These results imply that PA is an important contributor to children’s and adolescents’ mental health [13].

The coronavirus disease 2019 (COVID-19) pandemic has significantly changed the lives of children and adolescents worldwide. Due to the extensive measures implemented by many national governments to reduce the risk of person-to-person transmission of COVID-19, children and adolescents had to face social distancing rules, temporary school closures, and massive restrictions on their leisure activities. While these measures effectively slowed down the spread of the virus and contained the disease, they came with several negative consequences, including increased engagement in sedentary behavior [14] and substantially reduced PA [15, 16]. A survey among Canadian children and adolescents showed that only 3.6% of children and only 2.6% of adolescents were meeting the recommendation of 60 min of moderate-to-vigorous PA per day during the COVID-19 pandemic [17], down from the reported 12.7% meeting the guidelines before the pandemic [18]. In Germany, engagement in sports activities decreased by 6.5% and 14.6% among children and adolescents, respectively [19]. The reduced PA was accompanied by adverse changes in children’s physical fitness and increased BMI [20]. Overall, a meta-analysis of results from different countries revealed that, on average, child and adolescent engagement in total daily PA decreased by 20% during the pandemic [15].

Moreover, the COVID-19 pandemic had a strong negative impact on children’s and adolescents’ mental health. Under normal circumstances, the worldwide prevalence of mental disorders in this target group is 13.4% [21], which has, however, significantly increased during the pandemic. Existing reviews indicate an average doubling of clinically elevated anxiety (21%) and depression (25%) symptoms [22] and high pooled prevalences of depression (29%), anxiety (26%), sleep disorders (44%), and posttraumatic stress symptoms (48%) [23]. A representative survey in Germany found that two-thirds of children and adolescents reported being burdened by the pandemic and experienced significantly lower health-related quality of life (40% vs. 15%), more mental health problems (18% vs. 10%) and higher anxiety levels (24% vs. 15%) than before the pandemic [24]. These impairments in quality of life and mental health remained relatively stable during the pandemic [25].

Based on the aforementioned relationship between PA and mental health, researchers have suggested that higher engagement in PA may be an effective strategy for maintaining good mental health during the COVID-19 pandemic [26, 27] as well as physically and mentally recovering from the pandemic [20]. Indeed, higher levels of moderate-to-vigorous PA during the pandemic were associated with better health conditions [28], higher quality of life [29], and lower levels of insomnia, depressive symptoms, and anxiety symptoms among children and adolescents [30, 31]. Adolescent athletes who were restricted from doing sports worsened their mental health and health-related quality of life, yet these trends were reversed after the athletes were allowed to return to their sports [32]. Similarly, after returning to sports club participation, school-aged children improved their physical fitness and health-related quality of life [20].

The above research implies that increasing PA after the COVID-19 pandemic may alleviate the adverse effects the pandemic had on children’s and adolescents’ mental health. However, most of that research only measured self-reported PA, potentially limiting its implications. A recent study found that people were substantially biased when self-reporting their PA, with the level of overestimation being as high as 39% when compared with electronically measured PA [33]. Consequently, we need more objective data to validate the benefits of PA for physical and mental recovery from the pandemic. Furthermore, while the level of PA seems to keep increasing after lifting the COVID-19 restrictions [20, 32], it is unclear whether and how this increase has been affected by the pandemic. There is evidence that mental health before the pandemic predicted PA during the pandemic [34]. Consequently, it is also possible that mental health during the pandemic determines how fast children and adolescents return to their usual, before-pandemic PA levels after easing the restrictions. If the worsened mental health slowed the elevation of PA, we would need more intense PA promotion programs specifically targeted to this population.

The aim of this study, therefore, was to test the reciprocal relationship between PA and mental health in children and adolescents. Schools are widely recognized as essential for promoting PA in this target group [35], as the school setting provides many different opportunities for formal and informal PA, such as regular courses, active breaks between classes, or after-school programs [36, 37]. We thus sampled school-aged children and collected data on their mental health problems, health-related quality of life, and PA both before and during the easing of COVID-19 restrictions. We hypothesized that the level of PA would gradually increase with the easing of restrictions. We further hypothesized that mental health before the easing of COVID-19 restrictions would be associated with (the elevation of) PA during the easing of restrictions and that the level of PA would predict the improvement of mental health after the pandemic.

## Methods

### Participants and design

This was a prospective 6-week study with two measurement occasions (waves) conducted in a Bavarian elementary and grammar school in Munich, Germany. Participants were elementary (ranging from grade 1 to grade 4) and grammar school (ranging from grade 5 to 11) students aged 6 to 18 years. Both schools were part of a larger all-day campus offering regular PA in various areas of everyday school life, such as during recess periods, regular courses, active breaks between classes, and after-school programs. In particular, the curriculum included daily physical education lessons for all grades, ranging from 60 to 80 min and 90 to 120 min for the elementary and grammar school, respectively. All 322 students (215 elementary school students and 107 grammar school students) were approached by the experimenter and asked to participate in the study. Of them, 170 individuals (71 elementary school students and 99 grammar school students) volunteered to participate and signed informed consent. All 170 participants also completed the baseline and follow-up questionnaires. Inclusion criteria were proficiency in German or English, using a smartphone or a smartwatch with a built-in pedometer, and signed informed consent from students and both legal guardians. For elementary school students, participation in the study also required their legal guardians to assist in filling in the questionnaires.

Pretest data collection for this study was undertaken in February 2022, shortly before the authorities eased and lifted the COVID-19 restrictions (March 1, 2022). The follow-up (posttest) data collection occurred six weeks later (April 2022). We chose the 6-week period based on previous studies on PA and mental health that typically range from four to twelve weeks [38], with most studies having a posttest after six weeks [39, 40]. Moreover, prior research revealed that a 6-week study period is sufficient to show significant effects [39]. The study protocol was approved by the Ethical Committee of the Saarland Medical Association (No. 52/22) and Bavarian State Medical Association (No. mb22069), and the Commissioner for Data Protection of the Saarland University. Participants were not compensated for their participation but could win a gift voucher at the study end.

### Procedure

Class teachers approached their students (or the students’ parents) and informed them about the study; the class teachers directly approached the grammar school students, whereas for elementary school students (aged 6 to 10 years), the teachers approached the students’ parents. Those willing to participate were then briefed in more detail on the study procedure by the first author and asked to provide written informed consent. Only students who voluntarily agreed to participate and whose parents signed informed consent were included in the study. A link to an online questionnaire measuring demographic variables, mental health problems, health-related quality of life, and self-reported PA was distributed by email in the first study week (pretest). A follow-up questionnaire on mental health problems, health-related quality of life, and self-reported PA was sent out six weeks later (posttest). Each time, participants logged in with their unique code. An email was sent to participants a few days before the follow-up to minimize attrition and to remind them to look out for the questionnaire in the following email. The questionnaires were programmed in the SoSci Survey software [41]. Due to possible difficulties in understanding for students in elementary school, parents of the elementary school participants were instructed to help their children complete the pretest and posttest questionnaires. The online measurements were necessary to not interfere with the school’s internal processes and workflows during the pandemic so that participants could complete the questionnaires from home. The actual PA was tracked electronically for six consecutive weeks with a built-in pedometer of the participants’ smartphones or smartwatches.

### Measures

#### Demographic variables

Participants indicated their age, gender, socioeconomic status, international background, and prior experience in exercise and sports. Socioeconomic status and international background were checked because children with international background and low socioeconomic status were affected significantly more by the COVID-19 pandemic [24]. Socioeconomic status was measured using the Family Affluence Scale (FAS III; [42]). The FAS III consists of six items regarding household wealth: number of cars (0, 1, 2 or more), number of bathrooms (0, 1, 2, 3 or more), number of computers (0, 1, 2, 3 or more), non-shared bedroom (no/yes), dishwasher (no/yes), and the number of holidays abroad during the last 12 months (0, 1, 2, 3 or more). The answers to the six items were summed up and could range from 0 to 13. Higher scores indicated a higher socioeconomic status of the participant’s family [43]. Prior experience in exercise and sports was measured by asking students whether they had participated in any organized exercise courses or played any organized sports before the COVID-19 pandemic.

#### Health-related quality of life

The KIDSCREEN-52 questionnaire [44] was used to assess health-related quality of life in children and adolescents. The questionnaire includes 52 items related to ten dimensions: (1) physical well-being, (2) psychological well-being, (3) moods and emotions, (4) self-perception, (5) autonomy, (6) the quality of parent relation and home life, (7) social support and peers, (8) the quality of school environment, (9) social acceptance, and (10) the satisfaction with financial resources. The items were presented with 5-point Likert-type scales to assess frequency (1 = never, 2 = seldom, 3 = quite often, 4 = very often, 5 = always) or intensity (1 = not at all, 2 = slightly, 3 = moderately, 4 = very, 5 = excessively). Item scores for each dimension were summed up for analysis. Higher scores indicated a better quality of life.

#### Mental health problems

Mental health was assessed using the Strengths and Difficulties Questionnaire (SDQ; [45]. The SDQ provides a total difficulty score across 20 items and four subscales regarding emotional problems, conduct problems, hyperactivity, and peer problems. Each problem scale consists of five items presented with three response options ranging from 0 (*not true*) to 2 (*certainly true*). Item scores for each subscale were summed up for analysis; higher scores indicated more difficulties. For elementary school students (aged 6 to 10 years), the parent SDQ was administered to measure the students’ mental health problems as reported by their parents. Usually, the SDQ also includes a prosocial behavior subscale, yet this subscale was not used because the study particularly focused on mental health problems [24, 25]. Both the self-reported and the parent-reported versions of the SDQ have been found to provide a valid and reliable measurement of mental health problems in children and adolescents [46, 47].

#### Physical activity

Physical activity (PA) was operationalized as the number of daily steps and measured electronically and continuously using smartwatches and/or smartphones with a built-in pedometer. Smart devices, both smartwatches and smartphones, used for monitoring PA (e.g., step counts) are increasingly popular, and the prevalence among children and adolescents in Germany is remarkable [48]. In particular, 66% of children aged 6 to 9, 92% of early adolescents aged 10 to 13, and 98% of adolescents aged 14 to 18 regularly use such gadgets to track and quantify their daily movement [49]. Researchers have found that smart devices are acceptable and commonly used methods in tracking activity behaviors in children [50] and adolescents [51], mainly because they are feasible and user-friendly [50–52]. Evidence also shows that, for estimating the number of steps in a real-life setting, smartphones and/or smartwatches are accurate and reliable tools [53, 54]. Due to the many available brands and fitness apps on the market, we accepted all devices that utilized pedometers and were designed to be worn continuously. Participants were asked to wear their smartwatch or have their smartphone with them all day long (except for sleeping). The mean number of daily steps per week was calculated for each of the consecutive six weeks and used for analysis as the primary physical activity variable. In addition, we administered a single frequency item *“Please indicate on how many days did you engage in sport or exercise in the last week”* [55] in the two online measurements (see Procedure) to additionally check for the evolution of PA from pretest to posttest.

### Statistical analyses

Descriptive analysis was used to describe the study sample. Linear multilevel growth models were computed to test whether PA (the number of daily steps) changed over time. The growth modeling was employed because of the hierarchical structure of our data, in which each time measurement is nested within each participant (Level 1 = weeks, Level 2 = participants). We did not include Level 3 (= elementary vs. grammar school) in the growth modeling because the preliminary intercept-only model revealed that school affiliation did not explain a significant portion of the variance in physical activity. Intercept-only models were first estimated to explore the degree of variance in PA attributable to the between- and within-person levels. Second, we computed an unconditional linear growth model for PA, containing only a “time” variable as a predictor, to examine its change over time. Third, we tested conditional growth models to control whether demographic variables had an effect on PA; predictor (fixed) variables consisted of intercept, time (week 1 to 6), and the five demographic variables (gender, age, international background, economic status, and prior experience in exercise and sports), and random effects included a random intercept and a random slope for “time”. The variance type “unstructured”, which fits all variances and covariances between random effects, was selected. Finally, to test the predictive effect of mental health problems and health-related quality of life on (the evolution of) PA, we computed separate conditional growth models in which the pretest SDQ and KIDSCREEN variables (and their interactions) with “time” were included as predictors. We controlled for international background and economic status in these models because they had significant effects on participants’ PA. There were very few missing data for participants’ PA (0.01%) and no missing data for the SDQ and KIDSCREEN variables and covariates; the cases with missing data were included in the analyses because mixed effects models can be reliably estimated in the presence of partially missing data.

To test whether mental health and health-related quality of life changed from pretest to posttest, paired *t*-tests were computed for the SDQ and KIDSCREEN variables. Hierarchical multiple regression analyses were then conducted on posttest SDQ and KIDSCREEN scores to test whether PA could predict the change in mental health. The respective pretest SDQ or KIDSCREEN score was entered as the first blocking variable, and PA (the mean number of daily steps) was added on the second step. Normal probability plots of the standardized residual and scatterplots of residuals were generated to test normality, linearity, and homoscedasticity. The non-autocorrelation assumption was also met (Durbin-Watson-test; 1.5 < d < 2.5 for all regression models). In addition, no serious multicollinearity problems among the predictor variables were found (all variance inflation factor statistics < 4.0). All analyses were performed with SPSS 27.0 (IBM Corp.; Armonk, NY). The level of significance was set at *p* < .05 (two-tailed). To graphically display the results, participants were dichotomized into low and high in a given SDQ and KIDSCREEN variable based on the German norm values.

### Power analysis

An a priori sample-size calculation with the G*Power software [56] for linear regression with two tested predictor variables (pretest, time), based on middle effect size (*f*^2^ = 0.15), power of 0.80, and significance level of 0.05, resulted in a minimal sample size of 89 participants. The medium effect size was based on prior research investigating exercise effects on mental health in children and adolescents [9]. Regarding multilevel growth models, researchers have shown that a sample of 50 or more participants is required at Level 2 to avoid biased estimates [57]; our sample at Level 2 included 170 participants.

## Results

### Sample characteristics

The sample comprised 170 elementary and grammar school students (Table 1). Most students self-identified their current gender as female (42%) or male (55%). 60 students (35%) reported having an international background. The household wealth of participants ranged from 4 to 13 (*M* = 9.84, *SD* = 2.13), indicating an average-to-high economic status of the participants’ families. Most participants (90%) reported past sport or exercise behavior. Students from 11 different classes were included in the sample; the inter-class distribution was approximately the same (15–20 students from each class) except for the two oldest classes from which only 5 and 6 students participated.

**Table 1:** Characteristics of the study sample (N = 170)

	Elementary school (*n* = 71)			Grammar school (*n* = 99)	
	*n* (%)	*M (SD)*		*n* (%)	*M (SD)*
Gender					
Men	32 (45)			62 (63)	
Women	38 (54)			33 (33)	
Diverse	1 (1)			4 (4)	
Age (years)		8.04 (1.20)			13.10 (2.10)
International background					
Yes	18 (25)			42 (42)	
No	53 (75)			57 (58)	
Economic status		9.34 (2.26)			10.20 (1.97)
Past sport/exercise behavior					
Yes	61 (86)			92 (93)	
No	10 (14)			7 (7)	

*Note*. Economic status can range from 0 to 13, with higher values representing higher status.

### Evolution of physical activity

Growth models revealed a significant linear effect of time, *F*(1, 169) = 21.94, *p* < .001, indicating an increase in physical activity with each subsequent week over the study period (Table 2), *b* = 100.00, *t*(169) = 4.68, *p* < .001, 95% CI [57.85, 142.15]. There were also significant effects of international background, *F*(1, 163) = 7.90, *p* = .006, and economic status, *F*(1, 163) = 5.59, *p* = .019. Participants with international background were less physically active than their peers, *b* = -607.90, *t*(163) = -2.81, *p* = .006, 95% CI [-1,035.08, -180.02], while economic status positively predicted physical activity, *b* = 117.15, *t*(163) = 2.37, *p* = .019, 95% CI [19.32, 214.98]. The analysis further revealed significant variance in intercepts across participants, Var(*u*_*0j*_) = 1,493,960, *Z* = 18.44, *p* < .001, and in the slopes across participants, Var(*u*_*1j*_) = 27,022, *Z* = 3.05, *p* = .002. The covariance between intercept and slope was not significant, indicating no between-participant variation in how physically active they became over time. Participants’ age, gender, and past sport or exercise behavior did not predict PA. Self-reported days of sport or exercise behavior increased from pretest (*M* = 4.54, *SD* = 1.52) to posttest (*M* = 5.54, *SD* = 1.26), *t*(169) = -7.96, *p* < .001, Cohen’s *d*_z_ = 0.61, supporting the aforementioned growth model results that participants enhanced their PA over the 6-week study period.

**Table 2:** Means (and *SD*s) of daily steps during the six weeks

	Elementary school (*n* = 71)	Grammar school (*n* = 99)	Total sample (*n* = 170)
Week 1	6701 (593)	7454 (1557)	7139 (1230)
Week 2	7295 (711)	7506 (1931)	7417 (1544)
Week 3	7386 (841)	7483 (1861)	7443 (1518)
Week 4	7411 (790)	7682 (1900)	7569 (1539)
Week 5	7500 (660)	7805 (2286)	7678 (1798)
Week 6	7367 (674)	7866 (2177)	7658 (1731)

#### The effect of mental health problems on physical activity

**Fig. 1:**
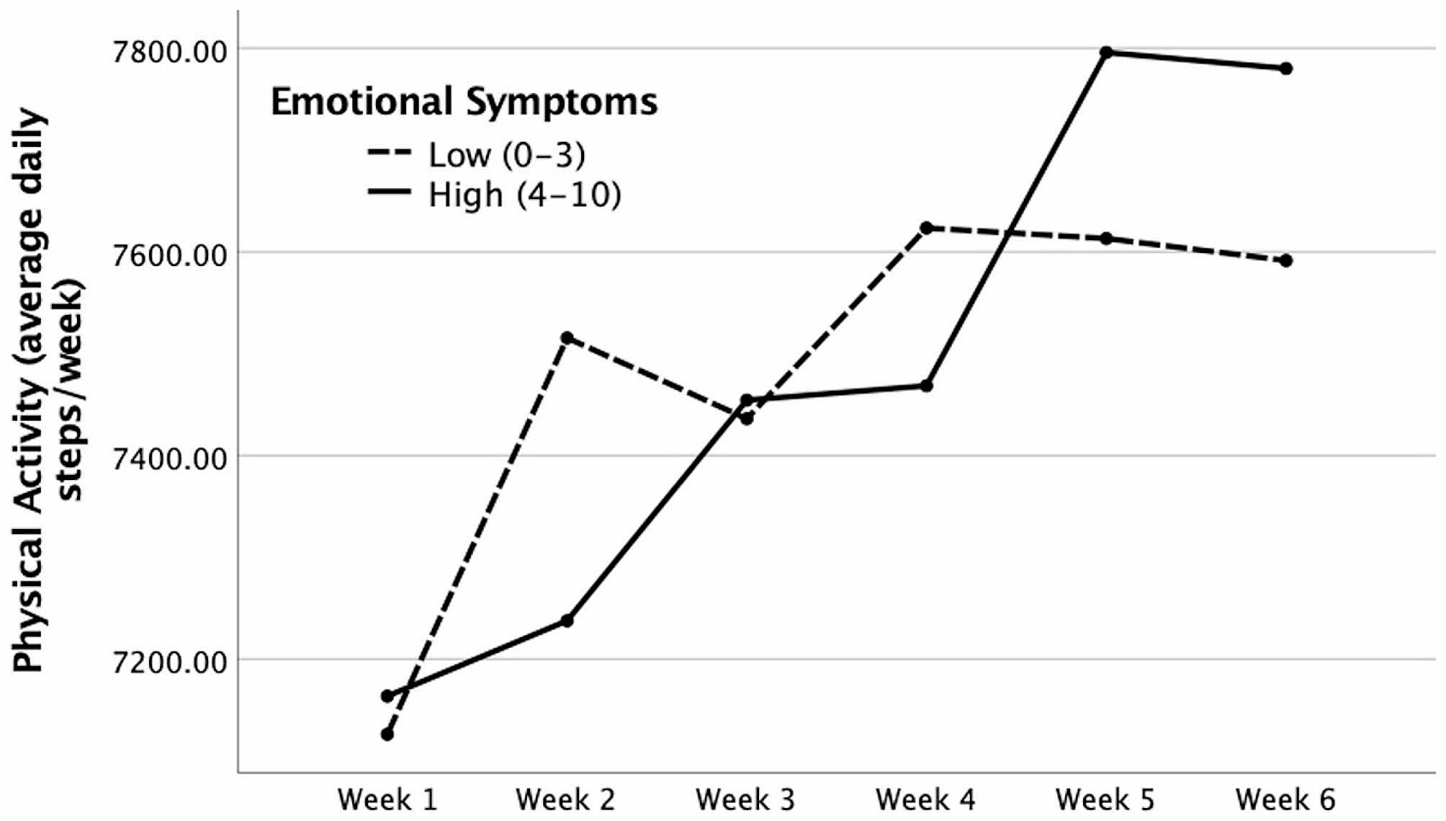
Physical activity of participants with high and low levels of emotional symptoms

Mental health problems did not predict how participants changed their PA over the 6-week period (Table 3), except for the Emotional Symptoms variable, which showed a significant interaction with Time, *b* = 18.44, *t*(168) = 2.06, *p* = .041, 95% CI [0.76, 36.11]. Participants with higher levels of emotional symptoms showed a sharper increase in PA towards the end of the 6-week period than participants with lower levels of emotional symptoms (see Fig. 1). We found no main effect for any of the SDQ variables (Table of main effects for the SDQ variables: Additional file 1).

**Table 3:** Parameter estimates (unstandardized) for the growth models examining the relationship between mental health problems and the evolution of physical activity during the COVID-19 pandemic

	Mental health problems (total)	Emotional symptoms	Conduct problems	Hyperactivity	Peer problems
Fixed effects					
Intercept	6,204.32^**^	6,293.37^**^	5,838.16^**^	6,161.62^**^	6,091.51^**^
International background	-650.81^**^	-668.41^**^	-645.25^**^	-645.51^**^	-648.25^**^
Economic status	124.72^**^	125.49^**^	127.96^**^	125.77^**^	124.74^**^
Time	82.57	44.59	97.17^**^	129.29^**^	110.40^**^
Variable	-13.61	-79.28	79.72	-32.86	-18.68
Time × Variable	1.57	**18.44** ^*^	1.33	-7.69	-4.87
Random effects					
Residual	883,275^**^	883,275^**^	883,275^**^	883,275^**^	883,275^**^
Intercept	1,469,071^**^	1,441,107^**^	1,455,043^**^	1,468,251^**^	1,473,954^**^
Slope	27,402^**^	25,564^**^	27,479^**^	27,155^**^	27,418^**^
Cov (Intercept, Slope)	-41,146	-33,914	-41,831	-43,073	-42,048

*Note*. Cov = covariance. The dependent variable was physical activity (number of daily steps) during the six weeks. International background: 0 = no, 1 = yes. Significant relationships between mental health problems and physical activity are marked bold.

^*^p < .05; ^**^p < .01

#### The effect of health-related quality of life on physical activity

Health-related quality of life at the pretest was largely unrelated to the evolution of PA over the 6-week period. None of the health-related quality of life variables predicted how participants changed their PA over time, as indicated by non-significant Variable × Time interactions (Table 4). We only found a main effect of physical well-being on PA, *b* = 85.52, *t*(166) = 2.97, *p* = .003, 95% CI [28.61, 142.44], indicating that participants with higher physical well-being were generally more active (Table of main effects for the KIDSCREEN variables: Additional file 2).

**Table 4:** Parameter estimates (unstandardized) for the growth models examining the relationship between health-related quality of life and the evolution of physical activity during the COVID-19 pandemic

	Physical well-being	Psychological well-being	Moods and emotion	Self-perception	Autonomy	Parent relation	Financial resources	Social support	School environment		Social acceptance	
Fixed effects												
Intercept	4,763.05^**^	5,961.54^**^	6,050.51^**^	5,929.09^**^	6,440.30^**^	5,524.23^**^	6,076.54^**^	5,312.46^**^	6,926.77^**^	5,163.92^**^		
International background	-712.19^**^	-663.84^**^	-643.09^**^	-642.22^**^	-632.56^**^	-645.18^**^	-616.43^**^	-635.78^**^	-585.00^**^	-631.94^**^		
Economic status	121.73^**^	127.40^**^	124.70^**^	124.90^**^	123.93^**^	124.90^**^	132.49^**^	127.59^**^	115.83^*^	121.32^*^		
Time	50.00	22.98	89.81	143.53	-29.71	265.75^*^	288.20^*^	58.46	-23.57	190.37		
Variable	**79.14** ^*^	2.84	-0.06	5.90	-20.88	21.66	-11.41	30.57	-35.18	70.56		
Time × Variable	2.89	3.32	0.37	-2.20	6.97	-6.86	-14.80	1.83	5.42	-6.92		
Random effects												
Residual	883,275^**^	883,275^**^	883,275^**^	883,275^**^	883,275^**^	883,275^**^	883,275^**^	883,275^**^	883,275^**^	883,275^**^		
Intercept	1,413,883^**^	1,475,482^**^	1,474,484^**^	1,473,635^**^	1,468,187^**^	1,466,621^**^	1,469,447^**^	1,453,424^**^	1,444,695^**^	1,449,069^**^		
Slope	27,393^**^	27,177^**^	27,479^**^	27,384^**^	26,863^**^	26,682^**^	26,114^**^	27,415^**^	26,827^**^	27,242^**^		
Cov (Intercept, Slope)	-45,832	-42,214	-41,687	-41,390	-39,658	-39,189	-41,673	-42,503	-36,774	-39,199		

*Note*. Cov = covariance. The dependent variable was physical activity (number of daily steps) during the six weeks. International background: 0 = no, 1 = yes. Significant relationships between health-related quality of life and physical activity are marked bold.

^*^p < .05; ^**^p < .01

### Evolution of mental health problems and health-related quality of life

Mental health problems slightly decreased from pretest to posttest, yet this change fell short of significance. Regarding single subscales, only hyperactivity was significantly reduced at the posttest. Health-related quality of life remained relatively stable, except for physical well-being, autonomy, and the quality of school environment, all of which were significantly improved at the posttest (Table 5).

**Table 5:** Mental health problems and health-related quality of life at the pretest and the posttest

	Pretest	Posttest	*t*-test (*df* = 169)	*p*-value	Cohen’s *d*_z_	
***Mental health problems***						
Mental health problems (total)	11.08 ± 5.72	10.48 ± 6.55	1.88	0.062	0.14	
Emotional symptoms	3.01 ± 2.37	2.90 ± 2.64	0.78	0.436	0.06	
Conduct problems	2.14 ± 1.70	1.96 ± 1.74	1.65	0.100	0.13	
Hyperactivity	3.81 ± 2.35	3.47 ± 2.31	2.90	**0.004**	0.22	
Peer problems	2.14 ± 1.66	2.15 ± 1.83	-0.11	0.916	0.01	
***Health-related quality of life***						
Physical well-being	17.29 ± 3.29	17.86 ± 3.40	-2.92	**0.004**	0.22	
Psychological well-being	23.21 ± 5.26	23.69 ± 4.76	-1.77	0.079	0.14	
Moods and emotion	27.53 ± 6.01	27.61 ± 6.20	-0.24	0.814	0.02	
Self-perception	19.76 ± 4.51	19.82 ± 4.95	-0.28	0.778	0.02	
Autonomy	18.61 ± 3.56	19.94 ± 3.43	-5.83	**0.000**	0.48	
Parent relation	24.16 ± 4.11	24.15 ± 4.37	0.21	0.983	0.00	
Financial resources	12.72 ± 2.49	12.71 ± 2.57	0.04	0.972	0.00	
Social support	22.72 ± 4.54	22.73 ± 4.36	-0.04	0.969	0.00	
School environment	22.79 ± 4.71	23.59 ± 4.76	-2.69	.**008**	0.21	
Social acceptance	13.05 ± 2.24	13.03 ± 2.48	0.14	0.891	0.01	

*Note*: Data are presented as mean ± SD. Significant *p*-values are marked bold. Cohen’s *d*_z_ is the measure of effect size. By convention, effect sizes of 0.20, 0.50, and 0.80 are considered small, medium, and large, respectively.

#### The effect of physical activity on mental health problems

Multiple regression analyses revealed no effect of physical activity on the reduction of mental health problems from pretest to posttest (see Table 6).

**Table 6:** Multiple regressions analysis of physical activity on the reduction of mental health problems (posttest)

	Mental health problems (total)			Emotional symptoms			Conduct problems			Hyperactivity			Peer problems	
	ß	*t*		ß	*t*		ß	*t*		ß	*t*		ß	*t*
***Step 1***														
Pretest variable	0.78	16.07^**^		0.76	14.98^**^		0.70	12.54^**^		0.79	16.73^**^		0.66	11.22^**^
***Step 2***														
Pretest variable	0.78	16.16^**^		0.76	14.98^**^		0.69	12.38^**^		0.79	16.67^**^		0.66	11.28^**^
Physical activity	0.07	1.47		0.04	0.72		0.09	1.68		0.03	0.52		0.08	1.38

*Note*. The dependent variables were the posttest SDQ variables, while controlling for the respective pretest SDQ variable. Standardized regression weights (ß) and the respective *t*-tests are reported.

^*^p < .05; ^**^p < .01

#### The effect of physical activity on health-related quality of life

Similarly, multiple regression analyses showed no effect of physical activity on the change in health-related quality of life (Table 7).

**Table 7:** Multiple regressions analysis of physical activity on the change in health-related quality of life (posttest)

	Physical well-being		Psychological well-being		Moods and emotion		Self-perception		Autonomy		Parent relation		Financial resources		Social support		School environment		Social acceptance	
	ß	*t*	ß	*t*	ß	*t*	ß	*t*	ß	*t*	ß	*t*	ß	*t*	ß	*t*	ß	*t*	ß	*t*
***Step 1***																				
Pretest variable	0.71	13.07^**^	0.76	15.06^**^	0.76	15.23^**^	0.80	17.52^**^	0.64	10.78^**^	0.65	11.04^**^	0.62	10.24^**^	0.62	10.21^**^	0.66	11.35^**^	0.56	8.64^**^
***Step 2***																				
Pretest variable	0.72	12.92^**^	0.76	15.12^**^	0.77	15.37^**^	0.81	17.57^**^	0.64	10.71^**^	0.65	11.01^**^	0.61	10.10^**^	0.63	10.39^**^	0.67	11.40^**^	0.56	8.69^**^
Physical activity	− 0.04	-0.65	0.07	1.43	0.08	1.68	0.05	1.17	− 0.03	-0.50	− 0.03	-0.57	− 0.08	-1.26	− 0.10	-1.67	0.07	1.11	− 0.06	-0.95

*Note*. The dependent variables were the posttest KIDSCREEN variables, while controlling for the respective pretest KIDSCREEN variable. Standardized regression weights (ß) and the respective *t*-tests are reported.

^*^p < .05; ^**^p < .01

## Discussion

This study aimed to test the reciprocal relationship between PA and mental health in German children and adolescents during the easing of COVID-19 restrictions. Pretest data collection for this study was undertaken in February 2022, shortly before the authorities eased and lifted the COVID-19 restrictions. The follow-up (posttest) data collection occurred six weeks later (April 2022), while PA was measured continuously during the whole study period. In line with our hypothesis, we found that the level of PA gradually increased after the easing of restrictions. This increase was unrelated to pretest mental health problems and health-related quality of life except for emotional symptoms. In particular, participants with higher emotional problems engaged less in PA shortly after the easing of restrictions but became more physically active towards the end of the study. We further found that hyperactivity decreased and physical well-being, perceived autonomy, and perceived quality of the school environment improved after the easing of COVID-19 restrictions. However, none of these changes was predicted by the level of participants’ PA.

The finding that PA increased after the COVID-19 restrictions had been eased is in line with studies that compared children’s and adolescents’ PA upon return to school [58, 59]. This is not surprising given that COVID-19 restrictions removed many opportunities for children and adolescents to be active, such as active travel to school, school break times, and sports activities in and out of the school context [36, 37]. Furthermore, our analysis revealed no covariance between intercept and slope in the elevation of PA, indicating that the participants became more physically active after the pandemic regardless of how active (or inactive) they were during the pandemic. The self-reported levels of PA also supported these results. The adverse effects of COVID-19 containment on PA thus seem to alleviate after children and adolescents are allowed to return to schools and organized leisure time activities.

Based on the evidence that mental health before the COVID-19 pandemic predicted PA during the pandemic [34], we hypothesized that mental health problems and health-related quality of life during the pandemic would determine how fast or slow participants would increase their PA levels after easing the COVID-19 restrictions. However, we did not find much support for this hypothesis. Pretest conduct problems, hyperactivity, peer problems, and overall mental health problems were unrelated to how PA changed over the study period. Only the level of emotional symptoms showed an interaction effect. Regarding the health-related quality of life, none of the KIDSCREEN variables predicted the elevation of PA after easing COVID-19 restrictions. These results indicate that the reasons for the gradually increased PA were largely unrelated to participants’ mental health. The mere return to school and the re-opening of sport clubs presumably had much stronger effects during this specific period and, in turn, suppressed the impact that mental health typically has on students’ engagement in PA behavior.

The interaction effect of emotional symptoms deserves mention. We found that participants who felt more anxious and worried engaged less in PA shortly after the easing of COVID-19 restrictions, yet they caught up and became more physically active towards the study end. On the one side, this may be an artifact due to multiple testing; indeed, the effect disappeared after the Bonferroni correction had been applied. On the other side, the result may be explained by higher initial doubts about, and slower adjusting to, the easing of COVID-19 restrictions among highly anxious participants, as also demonstrated in recent research [60]. Even though there were between 20 to ~ 200 daily steps/week differences among groups at each measurement point, this effect may be considered meaningful, as it demonstrates how even a small change in lifestyle towards more physical exercise can improve emotional well-being in young people. This increases the clinical relevance of this finding because more minor behavioral changes, in general, are easier to achieve than more considerable lifestyle alterations.

We further tested whether participants’ mental health changed after the easing of COVID-19 restrictions and whether higher engagement in PA would predict this change. Participants’ levels of hyperactivity decreased after the pandemic, while their physical well-being, perceived autonomy, and perceived quality of school environment improved, yet participants’ PA could predict none of these changes. This is at odds with previous studies that evidenced clear benefits of PA for children’s and adolescents’ mental health [1, 3, 5, 9]. A possible explanation is differences in study design and measurement of PA. Most previous studies involved a PA intervention, whereas we used an observational design. Moderate effects have been reported for intervention studies, while observational data show only weak or null associations [1, 5]. Furthermore, the majority of observational studies measured PA with self-reports, whereas we used a more objective behavioral measurement. The mere use of self-reported data often leads to overestimation [33] and the common method variance bias, which might have increased the strength of association in previous studies. An alternative, or additional, explanation is that the return to “normal” life after lifting the COVID-19 restrictions came along with several various positive changes that together had a substantial impact on improving people’s mental health and well-being, which made the typical positive effect of PA weaker and less visible. Indeed, researchers found that physically active children reported a better health-related quality of life than inactive children during COVID-19 lockdown, but these differences disappeared shortly after the lockdown. Moreover, the change in PA after the lockdown was unrelated to changes in health-related quality of life [58]. Hence, while lifting the COVID-19 restrictions brought benefits for children’s and adolescents’ mental health, these benefits were unrelated to the increase in PA, which might also be due to many other positive changes that emerged at the same time and together had a much more substantial impact.

Previous research found that boys are generally more physically active than girls and that PA declines with increasing age [61, 62]. However, as our results show, these differences might become attenuated during the COVID-19 pandemic. While some studies found that lockdown restrictions had a larger impact on PA in girls compared to boys and among older children [63], other researchers observed the disappearance of the typical sex difference in PA levels during the pandemic [59, 64], although these were re-established on return to school [58, 59]. Previous research also found that psychological resilience, which underlies mental health, increases with age due to cognitive development and more pronounced social interactions [65, 66]. This might have affected our results in terms of a stronger relationship between PA and mental health in children (elementary school students), as they are not as strong in resilience as adolescents (grammar school students) and thus more vulnerable to the effect of external factors such as physical activity. However, we did not find a moderator effect of age. The negative effect of lockdown restrictions on social interactions may explain this. Even though cognitive development, a promotive factor for resilience, is higher in adolescents, the adolescent students were at the same time much more burdened by social distancing and isolation during the pandemic, as they have stronger ties to friends outside the family when compared with younger children [65]. Consequently, it is possible that the positive effect of cognitive development and the negative effect of social isolation canceled each other, rendering no moderator effect of age in our study.

Regarding sample characteristics, as stated above, our study found no difference in PA between boys and girls and no effect of age. These results cannot be attributed to unequal variance between subgroups of participants, as we had a big enough sample of men and women and a similar number of participants from each class in the sample. Thus, our results do not allow for drawing sex- and age-based implications. However, we found significant effects of international background and economic status on PA. Participants with international background were less physically active than their peers, while economic status positively predicted physical activity. This is in line with a recent nationwide study in Germany that children with low socioeconomic status and international background were affected significantly more by the pandemic [24]. Consequently, these groups might need specific COVID-recovery interventions.

### Strengths and limitations

The present study has multiple strengths, including objectively measured PA with pedometers, a prospective research design, and high statistical power to detect predicted effects. These objectively measured results confirm and extend the findings of previous studies conducted during the easing of the COVID-19 restrictions. However, this study also has several limitations. First, we used a convenience sample from one large school in Bavaria, Germany. This population need not be representative of the general German population. Second, especially in young children (< 10 years), questions on mental health problems and health-related quality of life might be challenging to interpret or answer. However, parents were instructed to complete the questionnaire with their child to prevent bias. Third, although participants provided an almost complete dataset (99.9%) on their daily PA for the analysis, we could not check whether participants had worn the pedometers during their whole active daytime. Fourth, the Hawthorne effect could compromise the internal validity of our study, as participants were aware of being in a study and might have thus increased their PA in order to conform with what was expected from them [67]. Finally, seasonal influences may have affected the results, as the easing of COVID-19 restrictions, and thus also our study, started at the end of winter, and the data collection continued in the early springtime. The findings thus cannot be overstated. However, given similar results from studies that tested PA after schools re-open in different periods [58, 59, 64], the seasonal effect seems unlikely to account for the study results alone.

## Conclusion

In sum, this study found that children’s and adolescents’ PA increased and mental health improved after the authorities eased the COVID-19 restrictions. However, these effects were largely unrelated. Participants’ mental health before the easing did not predict how fast or slow the participants re-engaged in PA after easing the restrictions. Similarly, engagement in PA after lifting the restrictions did not significantly contribute to the participants’ improvement of mental health and health-related quality of life in this sample during a relatively short observational period. Future studies on the interrelations between PA and mental health in children and adolescents are warranted.

### Electronic supplementary material

Below is the link to the electronic supplementary material.

Supplementary Material 1: Main effects of the SDQ variables on physical activity.

Supplementary Material 2: Main effects of the KIDSCREEN variables on physical activity.

## Data Availability

The datasets generated and/or analyzed during the current study are available in the figshare repository, 10.6084/m9.figshare.23295872.v1.

## Abbreviations

b: Beta coefficient (coefficient estimate)
CI: Confidence interval
Cohen’s dz: Effect size used to indicate the standardised difference between two means
Cov: Covariance (of slopes and intercepts)
COVID-19: Coronavirus disease 2019
f²: Effect size
FAS III: Family Affluence Scale III
KIDSCREEN: KIDSCREEN-52 questionnaire
M: Mean
min: Minutes
N: Number of samples to be tested
n: Number of participants in the sample
p: Probability value (p-value)
PA: Physical Activity
SD: Standard deviation
SDQ: Strengths and Difficulties Questionnaire
t: T-test
Var(u0j): Variance of intercepts
Var(u1j): Variance of the slops
Z: Wald statistic (standard z-score)
-2LL: -2 Log-likelihood
