# Supplementary material for: Physical activity and mental health in school-aged children: a prospective two-wave study during the easing of the COVID-19 restrictions
Source: Child Adolesc Psychiatry Ment Health. 2024 Jan 3;18:4. doi: 10.1186/s13034-023-00695-8 (PMC10765890; doi:10.1186/s13034-023-00695-8)
Supplement: Supplementary file 1 — Supplementary Material 1: Main effects of the SDQ variables on physical activity. [file 13034_2023_695_MOESM1_ESM.docx]

**Additional file 2** Parameter estimates (unstandardized) for the growth models examining the main effect of health-related quality of life on physical activity during the COVID-19 pandemic

|  | **Physical well-being** | **Psychological well-being** | **Moods and emotion** | **Self-perception** | **Autonomy** | **Parent relation** | **Financial resources** | **Social support** | **School environment** | | **Social acceptance** | |
| --- | --- | --- | --- | --- | --- | --- | --- | --- | --- | --- | --- | --- |
| Fixed effects |  |  |  |  |  |  |  |  |  |  | |  |
| Intercept | 4,652.57^**^ | 5,794.51^**^ | 6,028.56^**^ | 6,022.78^**^ | 6,162.14^**^ | 5,879.48^**^ | 6,489.02^**^ | 5,222.48^**^ | 6,666.23^**^ | 5,356.23^**^ | |  |
| International background | -712.19^**^ | -663.84^**^ | -643.09^**^ | -642.22^**^ | -632.56^**^ | -645.18^**^ | -616.43^**^ | -635.78^**^ | -585.00^**^ | -631.94^**^ | |  |
| Economic status | 121.73^**^ | 127.40^**^ | 124.70^**^ | 124.90^**^ | 123.93^**^ | 124.90^**^ | 132.49^**^ | 127.59^**^ | 115.83^*^ | 121.32^*^ | |  |
| Time | 100.00^**^ | 100.00^**^ | 100.00^**^ | 100.00^**^ | 100.00^**^ | 100.00^**^ | 100.00^**^ | 100.00^**^ | 100.00^**^ | 100.00^**^ | |  |
| Variable | **85.52^**^** | 10.04 | 0.74 | 1.16 | -5.93 | 6.96 | -43.84 | 34.53 | -23.75 | 55.83 | |  |
| Random effects |  |  |  |  |  |  |  |  |  |  | |  |
| Residual | 883,275^**^ | 883,275^**^ | 883,275^**^ | 883,275^**^ | 883,275^**^ | 883,275^**^ | 883,275^**^ | 883,275^**^ | 883,275^**^ | 883,275^**^ | |  |
| Intercept | 1,412,076^**^ | 1,474,754^**^ | 1,472,368^**^ | 1,471,959^**^ | 1,468,919^**^ | 1,468,184^**^ | 1,473,809^**^ | 1,451,585^**^ | 1,445,564^**^ | 1,448,079^**^ | |  |
| Slope | 27,022^**^ | 27,022^**^ | 27,022^**^ | 27,022^**^ | 27,022^**^ | 27,022^**^ | 27,022^**^ | 27,022^**^ | 27,022^**^ | 27,022^**^ | |  |
| Cov (Intercept, Slope) | -45,013 | -41,879 | -40,705 | -40,611 | -39,999 | -39,917 | -43,663 | -41,653 | -37,187 | -38,733 | |  |

*Note*. Cov = covariance. The dependent variable was physical activity (number of daily steps) during the six weeks. In this model, only the main effect of the respective KIDSCREEN variable on PA was evaluated. International background: 0 = no, 1 = yes. Significant relationships between health-related quality of life and physical activity are marked bold.

^*^*p* < .05; ^**^*p* < .01
