# Supplementary material for: Physical activity and mental health in school-aged children: a prospective two-wave study during the easing of the COVID-19 restrictions
Source: Child Adolesc Psychiatry Ment Health. 2024 Jan 3;18:4. doi: 10.1186/s13034-023-00695-8 (PMC10765890; doi:10.1186/s13034-023-00695-8)
Supplement: Supplementary file 2 — Supplementary Material 2: Main effects of the KIDSCREEN variables on physical activity. [file 13034_2023_695_MOESM2_ESM.docx]

**Additional file 1** Parameter estimates (unstandardized) for the growth models examining the main effect of mental health problems on physical activity during the COVID-19 pandemic

|  | **Mental health problems (total)** | **Emotional symptoms** | **Conduct problems** | **Hyperactivity** | **Peer problems** |
| --- | --- | --- | --- | --- | --- |
| Fixed effects |  |  |  |  |  |
| Intercept | 6,166.86^**^ | 6,176.69^**^ | 5,832.06^**^ | 6,225.47^**^ | 6,113.98^**^ |
| International background | -650.81^**^ | -668.41^**^ | -645.25^**^ | -645.51^**^ | -648.25^**^ |
| Economic status | 124.72^**^ | 125.49^**^ | 127.96^**^ | 125.78^**^ | 124.74^**^ |
| Time | 100.00^**^ | 100.00^**^ | 100.00^**^ | 100.00^**^ | 100.00^**^ |
| Variable | -10.23 | -40.48 | 82.58 | -49.64 | -29.20 |
| Random effects |  |  |  |  |  |
| Residual | 883,275^**^ | 883,275^**^ | 883,275^**^ | 883,275^**^ | 883,275^**^ |
| Intercept | 1,467,317^**^ | 1,447,572^**^ | 1,452,924^**^ | 1,467,620^**^ | 1,472,110^**^ |
| Slope | 27,022^**^ | 27,022^**^ | 27,022^**^ | 27,022^**^ | 27,022^**^ |
| Cov (Intercept, Slope) | -40,330 | -38,985 | -40,848 | -42,784 | -41,195 |

*Note*. Cov = covariance. The dependent variable was physical activity (number of daily steps) during the six weeks. In this model, only the main effect of the respective SDQ variable on PA was evaluated. International background: 0 = no, 1 = yes.

^*^*p* < .05; ^**^*p* < .01
